# A comparison between topical and retrobulbar anesthesia in 27-gauge vitrectomy for vitreous floaters: a randomized controlled trial

**DOI:** 10.1186/s12886-018-0838-7

**Published:** 2018-07-07

**Authors:** Rong Han Wu, Rui Zhang, Zhong Lin, Qi Hua Liang, Nived Moonasar

**Affiliations:** 1 The Eye Hospital, School of Ophthalmology and Optometry, Wenzhou Medical University, No. 270 West College Road, Wenzhou, 325027 Zhejiang China; 2 Liaocheng People’s Hospital of Shandong Province, Liaocheng, Shandong China; 3 Caribbean Eye Institute, Valsayn, Trinidad and Tobago

**Keywords:** Topical anesthesia, Retrobulbar anesthesia, 27-gauge, Pars plana vitrectomy

## Abstract

**Background:**

To compare the safety and efficacy of topical anesthesia versus retrobulbar anesthesia in 27-gauge pars plana vitrectomy (PPV) for vitreous floaters.

**Methods:**

30 patients with vitreous floaters were randomized into Group T (topical anesthesia, proparacaine eye drop) and Group R (retrobulbar anesthesia), and underwent 27-gauge PPV. A 5-point visual analogue pain scale (VAPS) was used to assess patients’ pain experience of anesthesia and surgery procedure (during surgery, 2 h and 1 day after surgery).

**Results:**

The VAPS of anesthesia procedure was 1.27 ± 0.59 for patients in Group R, while it was all 0 for patients in Group T (*p* < 0.001). There was no significant difference for VAPS during surgery (Group T: 1.13 ± 0.74, Group R: 0.67 ± 0.62, *p* = 0.67), 2 h (Group T: 0.80 ± 1.01, Group R: 0.67 ± 0.62, *p* = 0.67) and 1 day (Group T: 0.20 ± 0.41, Group R: 0.27 ± 0.46, *p* = 0.68) after surgery between these two groups. Only one patient (6.7%) in Group T required additional topical anesthesia during the surgery. Most of the patients reported the pain experience came from initial trocar insertion in both groups. None of the patients required post operative analgesia in both groups. No intraoperative or postoperative complications were noted in both groups.

**Conclusion:**

This study suggested that topical anesthesia is a safe and effective anesthetic approach for patients with floaters who underwent 27-gauge PPV.

**Trial registration:**

ClinicalTrials.gov NCT03049163. Registered 8 February 2017.

**Electronic supplementary material:**

The online version of this article (10.1186/s12886-018-0838-7) contains supplementary material, which is available to authorized users.

## Background

The anesthetic methods for vitrectomy surgery include retrobulbar and peribulbar anesthesia. However, both methods have potential complications that can vary from minor to severe. For example, the complications of retrobulbar anesthesia include perforation of the ocular globe, retrobulbar hemorrhage, occlusion of the vein and/or the artery of the retina, retinal detachment, [1], subarachnoid injection, [2] intracranial diffusion, [3], cranial nerve palsies, [4], apnea and seizures [5].

Due to these potential complications, surgeons are trying to use simple and safe topical anesthesia to replace retrobulbar/peribulbar anesthesia. In a non-comparative study, Yepez et al. assessed the effect of topical anesthesia (4% lidocaine drops) combined with sedation in posterior vitrectomy procedures with various vitreoretinal diseases and found all patients had grade 1 (no) to grade 2 (mild) pain and discomfort during most of the procedure [6]. Besides, no patient required additional retrobulbar, peribulbar, or sub-Tenon’s anesthesia [6]. Later, several comparative studies directly compared the effect of topical anesthesia combined with sedation, or a series of steps of topical anesthesia to the retrobulbar/peribulbar anesthesia during posterior vitrectomy procedure, and consistently found the subjective pain scores were not significantly different [7–9].

Floaters are perceived by patients as a serious medical condition that has a significant negative impact on their vision and quality of life [10, 11]. Vitrectomy surgery for vitreous floaters is widely considered more straight forward than other vitreoretinal surgeries. Regarding the vitrectomy surgery procedure for vitreous floaters, it is much simpler than other vitreoretinal surgeries, mainly reflecting in lower usage of scleral indentation, photocoagulation, and an apparently shorter duration. Especially with regard to the use of 27-gauge vitrectomy, the sclerotomy is minimally invasive, and reduces the pain of surgery to some extent. Hence, retrobulbar anesthesia seemed excessive for this kind of surgery. Here, we compared the effect of topical and retrobulbar anesthesia for 27-gauge pars plana vitrectomy (PPV) for symptomatic vitreous floaters.

## Methods

### Subjects

Thirty eyes of 30 patients who underwent 27-gauge PPV for systemic vitreous floaters at the Eye Hospital of Wenzhou Medical University from March 2017 to July 2017 were alternatively randomized into Group T using topical anesthesia (15 eyes) or Group R using retrobulbar anesthesia (15 eyes). Both patients and surgeon were not blind to the randomizing result, since the patients would be aware of the anesthetic method when performing anesthesia and the surgeon could distinguish from the eye movement. The randomized order was produced by Excel randomized formula in advance by one of the author (ZL). Participants was enrolled and assigned to interventions by the same doctor (ZL). The inclusion criteria of this study were: (1) age > 18 years; (2) subjective sensation of the “floaters” which disturbed his/her life moderately or severely for more than 3 months; (3) clinical examination showed the vitreous opacity crumb; (4) patients who are willing to participate in this study. The exclusion criteria were (1): patients who had vitrectomy surgery before; (2) patients who had penetrating ocular trauma before; (3) patients with mental retardation, problem with communication, dementia or other systemic diseases that could not cooperate with this surgery.

All patients were informed about the purpose and nature of the study and underwent thorough preoperative counseling on what they would experience during surgery under topical or retrobulbar anesthesia; especially, being aware of the potential complications of retrobulbar anesthesia and some pain sensation in the eye if under topical anesthesia. This study adhered to CONSORT guidelines for reporting clinical trial. A completed CONSORT checklist is available in Additional file 1. The study protocol is available in https://clinicaltrials.gov.

Comprehensive preoperative ophthalmic examinations, including a slit lamp evaluation, best-corrected visual acuity (BCVA) in LogMar, intraocular pressure (IOP), B scan, optical coherent tomography, and fundus photography, were performed for patients preoperatively, 1 day and 1 week postoperatively. The study followed the tenets of the Declaration of Helsinki and was approved by the Ethics Committee of The Eye Hospital of Wenzhou Medical University. All patients signed informed consent forms.

### Surgery procedure

Before surgery, pupillary dilatation was obtained with 1% tropicamide. Retrobulbar anesthesia was achieved by injecting a 50% mixture of 2% lidocaine and 0.75% bupivacaine 4–5 ml through a 25 gauge (0.5 mm) needle. Topical anesthesia was performed by instilling 0.5% proparacaine hydrochloride (Alcaine®, Alcon, TX) 3 times (with 1 min-interval) before surgery. All retrobulbar anesthesia procedures were performed by the same doctor (ZL), while all 27-gauge PPV procedures were performed by the same surgeon (RHW). To begin the surgery, three transconjunctival sutureless 27-gauge cannulae (Constellation; Alcon Laboratories Inc., Fort Worth, TX), i.e. the inferior-temporal infusion cannula and the superior-nasal and superior-temporal operation cannulae, were made 4 mm posterior to the limbus with angled incision. Central vitreous followed by peripheral vitreous was removed with the help of a corneal contact lens. A quick scleral indentation was performed to check the extremely peripheral retina and ora serrata. Build-in laser (wavelength 532 nm) was used to perform the photocoagulation in eyes with lattice retinal degeneration or retinal break(s). At the end of the surgery, cannulae were removed and none of the eyes required suturing.

### Examinations and visual analogue pain scale (VAPS)

A 5-point visual analogue pain scale (VAPS), which was the main outcomes of this study, ranged from 0 (no pain) to 4 (severe pain), was used to assess the subjective pain experience of anesthesia and surgical procedure (during surgery, 2 h and 1 day after surgery). The exact painful surgery procedure would further ask if the patient felt pain during surgery. The 5-point visual analogue scale, ranged from 0 (extremely comfortable) to 4 (unable to perform surgery), was also used to assess the surgeon’s comfort and ease while performing the surgery [7–9]. Detailed information on the VAS was presented in Table 1. The detailed procedure of the pain experience during anesthesia and surgery was asked, if the patients reported a pain experience.

**Table 1:** Visual analogue scale for pain and surgeon’s comfort

Score	Pain	Surgeon’s comfort
0	No discomfort	Extremely comfortable
1	Mild discomfort	Mild movements/squeezing
2	Mild pain	Moderate discomfort (significant ocular movements/squeezing/Bells phenomenon)
3	Moderate pain	Severe discomfort hampering surgical maneuvering
4	Unbearable pain	Unable to perform surgery

#### Anesthesia procedure

If you felt discomfort/pain during the retrobulbar anesthesia, please choose the detailed procedure below (multiple choice).

A. topical eye drops instillation; B. needle puncture the skin; C. liquid injection; D. local pressure after injection; E. others (such as skin numbness, ptosis, lid swelling), please specify.

#### Surgery procedure

If you felt discomfort/pain during the surgery, please choose the detailed procedure below (multiple choice).

A. opened the lid using eye speculum; B. trocar inserted the sclera; C. vitrectomy; D. scleral indentation; E. cannula removal; F. others, please specify.

### Statistical analysis

All the data used and analyzed in this study can be accessed in Additional file 2. Student’s t-test or chi-square test was used for data comparison between the study groups. All statistical analysis was performed with Statistical Analysis System for Windows version 9.1.3 (SAS Inc., Cary, NC). A *P* value of < 0.05 was considered significant.

## Results

Fifteen eyes of 15 patients were included into Group T and Group R, respectively. The mean age of the total 30 patients was 32.4 ± 11.1 (range 19 to 51) years. There were 27 males (90%). The baseline characteristics of patients in these two anesthesia groups were presented in Table 2. There was no significant difference between age, gender, duration of floaters, preoperative BCVA and IOP between Group T and Group R.

**Table 2:** Baseline characteristics of patients in two anesthesia groups

	Group Topical	Group Retrobulbar	P
Age (year)^1^	32.9 ± 11.8	32.1 ± 10.8	0.85
Gender (male/female)^2^	13/2	14/1	1.00
Right/left eye^2^	8/7	10/5	0.71
Duration of floaters (month)^3^	36 (12, 60)	36 (18, 120)	0.45
BCVA (LogMar)^3^	0.0 (0.04, 0.0)	0.0 (0.05, 0.0)	0.28
IOP (mmHg)^1^	13.5 ± 2.7	15.9 ± 4.2	0.08

^1^presented as mean ± standard deviation, and tested by student’s t-test; ^2^ presented the number, and tested by Fisher Exact test; ^3^ presented as median and quartile range, and tested by Wilcoxon test

The mean surgery time of Group T and Group R was 14.0 ± 3.8 (range 9.2–18.6) and 13.6 ± 4.4 (range 8.7–19.1) minutes, respectively, while no significant difference was found (*p* = 0.45). One eye in each group was performed with photocoagulation (wavelength) because of retinal degeneration. However, none of these two patients required additional pain relief. The BCVA (LogMar) at 1 day (median 0.15 vs. 0.10 *p* = 0.85) and 1 week (median 0.0 vs. 0.0 *p* = 0.95) post-op were also not significantly different between the two groups. Similar results were found for IOP (1 day 8.4 ± 4.3 vs. 9.2 ± 3.9, p = 0.45; 1 week: 12.8 ± 3.6 vs. 14.6 ± 5.2. *p* = 0.29).

**Fig. 1:**
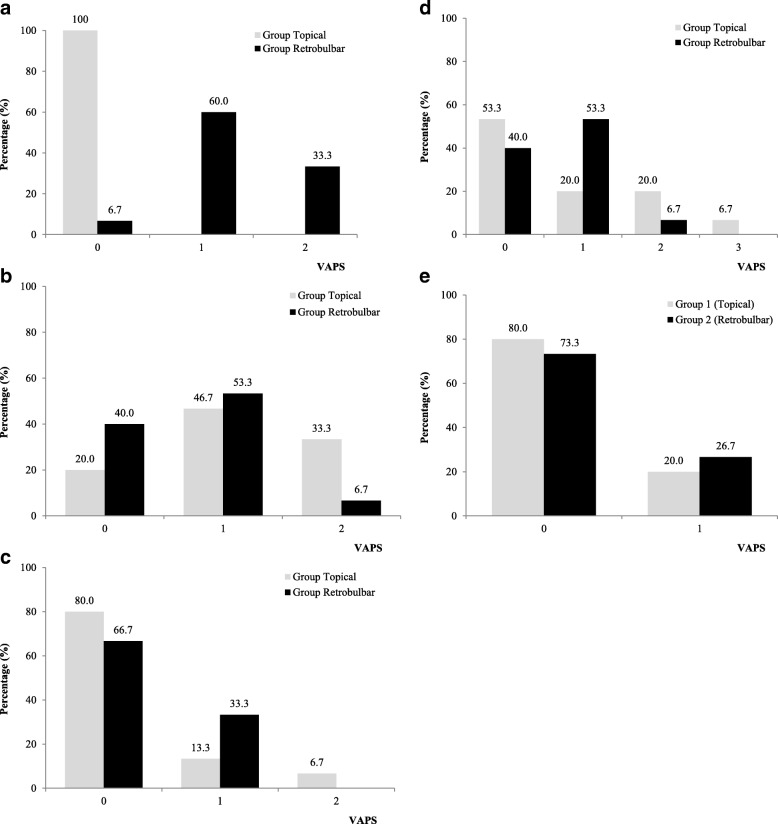
**a** Visual Analogue Pain Scale (VAPS) during anesthesia process. **b** Visual Analogue Pain Scale (VAPS) during surgery process. **c** Visual Analogue Scale (VAS) for surgeon’s comfort. **d** Visual Analogue Pain Scale (VAPS) 2 h after surgery. **e** Visual Analogue Pain Scale (VAPS) 1 day after surgery

The VAPS of anesthesia procedure was 1.27 ± 0.59 (range 0 to 2) for patients in Group R, while it was all 0 for patients in Group T (*p* < 0.001). The VAPS of surgery procedure was 1.13 ± 0.74 (range 0 to 2) and 0.67 ± 0.62 (range 0 to 2) for patients in Group T and Group R (*p* = 0.14), respectively. The VAP for surgeon’s comfort during the surgery was 0.27 ± 0.59 (range 0 to 2) and 0.33 ± 0.48 (range 0 to 1) for patients in Group T and Group R (*p* = 0.74), respectively. There was also no significant difference for VAPS 2 h (Group T: 0.80 ± 1.01, range 0 to 3, Group R: 0.67 ± 0.62, range 0 to 2, *p* = 0.67) and 1 day (Group T: 0.20 ± 0.41, range 0 to 1, Group R: 0.27 ± 0.46, range 0 to 1, *p* = 0.68) after surgery between these two groups. The distributions of the VAPS and VAS for surgeon’s comfort were presented in Fig. 1.

None of the patients in Group T felt discomfort or worse, while 14 patients felt discomfort or pain in Group R. Most of the patients reported that they experienced discomfort/pain when the needle punctured the skin (12/14, 85.7%), while a small proportion of patients reported a similar experience with the liquid injection (3/14, 21.4%) during the retrobulbar anesthesia. In Group T, 12 (80%) patients reported mild discomfort or worse during the surgery. Most of them reported the pain experience mainly came from initial trocar insertion (8/12, 67.7%) and use of the lid speculum (5/12, 41.7%), while a small proportion of patients reported that during scleral indentation (2/12, 16.7%), and vitrectomy procedure (1/12, 8.3%). In Group R, 9 (60%) patients reported mild discomfort or worse during the surgery. The patients reported the pain experience come from initial trocar insertion (3/9, 33.3%), use of the lid speculum (2/9, 22.2%), scleral indentation (2/9, 22.2%), vitrectomy procedure (1/9, 11.1%), and trocar removal (1/9, 11.1%). Only one patient (6.7%) in Group T required additional topical anesthesia (0.5% proparacaine hydrochloride eye drop, once) during the surgery. None of the patients required post operative pain relief in both groups. No intraoperative or postoperative complications were noted in both groups.

## Discussion

With development in techniques and technology, local anesthesia, including retrobulbar, peribulbar, and sub-Tenon’s anesthesia, is being used for the majority of vitreoretinal surgery. Although rare, many complications have been reported with injection anesthesia [1–5]. Topical anesthesia essentially eliminates the risk of needle-related complications associated with the injection of local anesthesia. Therefore, the safety and efficacy of the topical anesthesia have been investigated for small-gauge vitrectomy, and have been demonstrated to be safe and effective [8, 9, 12, 13]. However, to best of our knowledge, no study on the safety and efficacy of the topical anesthesia for 27-gauge PPV was reported.

There were several advantages in this study. First, this study was a randomized controlled trial, which may provide powerful evidence. Second, all of the surgeries and retrobulbar anesthesia of the study were performed by the same surgeon (RHW) and the same doctor (ZL), respectively, which may minimize the possible confounding factors, such as different pain experience during anesthesia or surgery with different techniques. Third, this study simplified the topical anesthesia procedure (only proparacaine hydrochloride eye drops for 3 times), compared to previous studies. Yepez et al. and Bahcecioglu et al. also used operative sedation for topical anesthesia patients [6, 7, 14]. Mahajan et al. used a serial topical anesthesia method, i.e., proparacaine hydrochloride drops, lignocaine gel for 1 min, another proparacaine hydrochloride infiltration with swab for 1 min [8]. Celiker et al. used proparacaine hydrochloride drops 15 min preceding surgery, and then proparacaine hydrochloride infiltration with sponges for another 15 min [9].

In this study, only eye drops were instilled for the topical anesthesia procedure, hence it was understandable that none of the patients felt uncomfortable or worse. The VAPS of surgery procedure in both groups ranged from 0 (no discomfort) to 2 (mild pain), and was not apparently different. The majority operative discomfort/pain experience reported by the patients was the trocar insertion, which was a very short period. Only one (6.7%) patient in Group T required additional topical anesthesia of eye drop, and also only one (6.7%) patient in Group T had significant eye squeezing that caused moderate discomfort for surgeon’s during the surgery, which suggested that most of the patients could tolerate the pain and cooperate well during the surgery. We believe the eye squeezing or movement could be conquered by detailed preoperative and operative communication with the patient, and by an experienced surgeon. Once the vitrectomy procedure is started, the movement of the eyeball can be controlled by surgeon with intraocular instruments.

Although slightly more proportion of patients (4/15, 26.7%) in Group T felt mild pain or moderate pain than that in Group R (1/15, 6.7%) 2 h after surgery, this was not significantly different. Besides, none of the patient required analgesic after surgery, suggesting a tolerable post-operation pain. Furthermore, the post-operation pain became negligible (no worse than mild discomfort) at day 1 post operation in both groups. All the data suggested that the topical anesthesia procedure was safe and efficient for the patients with floaters who underwent 27-gauge PPV.

Besides the most important advantage of topical anesthesia, i.e., eliminating the risk of needle-related complications, this anesthesia technique also greatly reduces the preparation time, eliminates the patients’ fear, has less interference to the post-operative recovery (such as lid edema, blink, eye movement, etc.), and has less surgical expenditure. The relatively small sample size and the fact that only patients with floaters were selected are some limitations to this study. For macular cases, such as macular holes and epimacular membranes, any inadvertent ocular movements might result in disastrous consequences. However, many surgeons have reported successful outcomes using topical anesthesia for such cases [6, 9, 13].

In summary, our study suggests that utilizing topical anesthesia (with only eye drops) is a safe and effective anesthesia approach for patients with floaters who underwent 27-gauge pars plana vitrectomy.

## Conclusions

The topical anesthesia is a safe and effective anesthesia approach for floaters removed by 27-gauge par plana vitrectomy, and could be recommended by clinical practice.

## Additional files

Additional file 1: CONSORT checklist. (XLSX 14 kb)

Additional file 2: Raw data used and analysed in this study. (XLSX 12 kb)

## Abbreviations

BCVA: Best-corrected visual acuity
IOP: Intraocular pressure
PPV: Pars plana vitrectomy
VAPS: Visual analogue pain scale

## References

[1] Vestal KP, Meyers SM, Zegarra H (1991). Retinal detachment as a complication of retrobulbar anesthesia. Canadian journal of ophthalmology.

[2] Ahn JC, Stanley JA (1987). Subarachnoid injection as a complication of retrobulbar anesthesia. Am J Ophthalmol.

[3] Marques-Gonzalez A, Onrubia-Fuertes X, Bellver-Romero J, Seller Losada JM, Pertusa-Collado V, Barbera-Alacreu M (1997). Intracranial diffusion. A complication of retrobulbar anesthesia. Revista espanola de anestesiologia y reanimacion.

[4] Jackson K, Vote D (1998). Multiple cranial nerve palsies complicating retrobulbar eye block. Anaesth Intensive Care.

[5] Moorthy SS, Zaffer R, Rodriguez S, Ksiazek S, Yee RD (2003). Apnea and seizures following retrobulbar local anesthetic injection. J Clin Anesth.

[6] Yepez J, Cedeno de Yepez J, Arevalo JF (2000). Topical anesthesia in posterior vitrectomy. Retina.

[7] Bahcecioglu H, Unal M, Artunay O, Rasier R, Sarici A (2007). Posterior vitrectomy under topical anesthesia. Canadian journal of ophthalmology Journal canadien d’ophtalmologie.

[8] Mahajan D, Sain S, Azad S, Arora T, Azad R (2013). Comparison of topical anesthesia and peribulbar anesthesia for 23-gauge vitrectomy without sedation. Retina.

[9] Celiker H, Karabas L, Sahin O (2014). A comparison of topical or retrobulbar anesthesia for 23-gauge posterior vitrectomy. J Ophthalmol.

[10] Zou H, Liu H, Xu X, Zhang X (2013). The impact of persistent visually disabling vitreous floaters on health status utility values. Quality of life research : an international journal of quality of life aspects of treatment, care and rehabilitation.

[11] Webb BF, Webb JR, Schroeder MC, North CS (2013). Prevalence of vitreous floaters in a community sample of smartphone users. International journal of ophthalmology.

[12] Theocharis IP, Alexandridou A, Tomic Z (2007). A two-year prospective study comparing lidocaine 2% jelly versus peribulbar anaesthesia for 25G and 23G sutureless vitrectomy. Graefe’s archive for clinical and experimental ophthalmology = Albrecht von Graefes Archiv fur klinische und experimentelle Ophthalmologie.

[13] Tang S, Lai P, Lai M, Zou Y, Li J, Li S (2007). Topical anesthesia in transconjunctival sutureless 25-gauge vitrectomy for macular-based disorders. Ophthalmologica Journal international d’ophtalmologie International journal of ophthalmology Zeitschrift fur Augenheilkunde.

[14] Yepez JB, de Yepez JC, Azar-Arevalo O, Arevalo JF (2002). Topical anesthesia with sedation in phacoemulsification and intraocular lens implantation combined with 2-port pars plana vitrectomy in 105 consecutive cases. Ophthalmic Surg Lasers.

